# The prevalence and outcomes of hyponatremia in children with COVID-19 and multisystem inflammatory syndrome in children (MIS-C)

**DOI:** 10.3389/fped.2023.1209587

**Published:** 2023-09-07

**Authors:** Neal Dalal, Mairead Pfaff, Layne Silver, Lily Glater-Welt, Christine Sethna, Pamela Singer, Laura Castellanos-Reyes, Abby Basalely

**Affiliations:** ^1^ Division of Nephrology, Department of Pediatrics, Cohen Children’s Medical Center of New York, New Hyde Park, NY, United States; ^2^ Division of Critical Care, Department of Pediatrics, Cohen Children’s Medical Center of New York, New Hyde Park, NY, United States; ^3^ Department of Pediatrics, Zucker School of Medicine at Hofstra/Northwell, Uniondale, NY, United States

**Keywords:** COVID-19, hyponatremia, MIS-C multisystem inflammatory syndrome in children, pediatric, prevalence, outcomes

## Abstract

**Introduction:**

To assess the prevalence of hyponatremia among pediatric patients with coronavirus disease 2019 (COVID-19) and Multisystem Inflammatory Syndrome in Children (MIS-C) and determine if pediatric hyponatremia was associated with an increased length of stay, higher rates of mechanical ventilation, and/or elevated inflammatory markers on admission as compared to eunatremic patients.

**Methods:**

Electronic health records were retrospectively analyzed for 168 children less than 18 years old with COVID-19 or MIS-C who were admitted to pediatric units within the Northwell Health system. The primary exposure was hyponatremic status (serum sodium <135 mEq/L) and the primary outcomes were length of stay, mechanical ventilation usage and increased inflammatory markers.

**Results:**

Of the 168 children in the study cohort, 95 (56%) were admitted for COVID-19 and 73 (43.5%) for MIS-C. Overall, 60 (35.7%) patients presented with hyponatremia on admission. Patients with hyponatremia had higher rates of intensive care unit admission when compared to eunatremic patients (32/60 [53.3%] vs. 39/108 [36.1%], *p* = 0.030). In regression models, hyponatremia was not significantly associated with increased length of stay or mechanical ventilation rates. After adjustment for relevant confounders, hyponatremia remained associated with an increased square root CRP (β = 1.79: 95% CI: 0.22–3.36) and lower albumin levels (β = −0.22: 95% CI: −0.42–−0.01).

**Conclusion:**

Hyponatremia is common in pediatric COVID-19 and MIS-C. Hyponatremia was associated with a lower albumin and higher square root CRP levels. This may suggest an association of inflammation with lower serum sodium levels.

## Introduction

As of May 2023, the United Stated reported 104 million cases of coronavirus disease 2019 (COVID-19); over four million of these cases occurred in children (1). Early on in the pandemic, the incidence of pediatric COVID-19 may have been significantly higher than reported due to the lack of widespread testing and decreased severity of disease in children as compared to adults (2–4). Only 1% of pediatric COVID-19 cases required hospitalization, however, 7.5% of these required pediatric intensive care unit admission (PICU) (5). Furthermore, the emergence of Multisystem Inflammatory Syndrome in Children (MIS-C), a serious disease associated with a recent COVID-19 infection involving inflammation of two or more organ systems, fever, severe illness, and epidemiologic evidence of SARS-CoV-2 infection, has posed increased risk to children (6–8). MIS-C is a dangerous and potentially fatal disease, with 68% of MIS-C cases requiring critical care support (9, 10). In addition to the morbidity above, in the pre-vaccine era, COVID-19 was reported to be associated with increased incidence of hyponatremia in adults (11).

Hyponatremia in critically ill adults and children has been shown to be an independent risk factor for mortality (12, 13). During the first wave of the pandemic, hyponatremia in COVID19 was found to be extremely prevelant and was associated with increased morbidity and mortality (12, 13). A prospective, observational, cohort study of adults with COVID 19 in Switzerland, found that COVID19 patients had higher incidence of hyponatremia compared to a control population of hospital patients who tested negative for SARS-CoV-2 (13). The exact etiology of hyponatremia in COVID-19 is unclear (14), however, inflammatory cytokines such as interlukein-1β and IL-6 induce secretion of arginine vasopressin which can cause the syndrome of inappropriate ADH secretion (SIADH) and thus hyponatremia. Disease severity was also correlated with sodium serum levels in adults with COVID-19 (14). Thus, hyponatremia may be a surrogate marker for disease severity or degree of inflammatory response (15). Moreover, hyponatremia was associated with a higher 30-day mortality rate, ICU admission, mechanical ventilation rates and length of stay (13).

While the associations of hyponatremia and COVID-19 is well established in adult populations, there are limited studies evaluating its prevalence and association with outcomes in pediatric patients (16, 17). In addition, while MIS-C is a new phenomenon, physicians have noted clinical similarities between MIS-C and Kawasaki disease (KD) (18). The severity of KD demonstrates strong association with hyponatremia and meta-analysis of MIS-C studies reported hyponatremia was present in 80.8% of MIS-C patients (19, 20). This meta-analysis aimed to determine the prevalence, outcomes and associated demographic and clinical variable in children with hyponatremia and COVID-19/MIS-C. We hypothesized that hyponatremia would be prevalent in pediatric patients with COVID-19 and MIS-C and would be associated with increased: length of stay (LOS), need for mechanical ventilation, and inflammatory markers at admission.

## Methods

### Patient population

A retrospective chart review of children less than 18 years old admitted to pediatric units within the Northwell Health system with acute COVID-19 or MIS-C was performed. Participant information was collected from hospitals within the New York metropolitan area; these included Cohen Children's Medical Center, an academic tertiary care children's hospital, as well as 3 tertiary hospitals: South Shore Hospital, Staten Island University Hospital, and Lenox Hill Hospital. Data from March 1, 2020, through February 28, 2021, was retrospectively collected using Sunrise Clinical Manager (Allscripts, Chicago, Illinois), an electronic health record system. The Institutional Review Board of Northwell Health approved this study.

Acute COVID-19 was defined as a positive polymerase chain reaction testing for severe acute respiratory syndrome coronavirus 2 (SARS-CoV-2) (by Northwell Health Labs) within 24 h of admission. MIS-C was defined by the Center for Disease Control and Prevention case definition: fever, significant evidence of inflammation, evidence of greater than or equal to 2 organ systems with dysfunction, and a positive test for current or recent SARS-CoV-2 infection or serological confirmation of exposure to COVID-19 within 4 weeks of symptom onset (21). Patients with hypernatremia (serum sodium >145 mEq/L on admission), pregnant patients, kidney transplant recipients, patients with kidney failure (estimated glomerular filtration rate <15 ml/min per 1.73 m^2^ or dialysis dependent), or those transferred from outside of the health system were excluded.

### Study variables

Demographic data including age, sex, self-reported race, and ethnicity, admission vital signs, laboratory values, in-hospital drug use, and echocardiogram data were extracted from medical records. Patient comorbidities including asthma, cancer, congestive heart failure, and immunosuppressed status were also collected.

### Exposure and outcomes

The primary exposure was hyponatremia, defined as a serum or plasma sodium level less than 135 mEq/L within 24 h of admission. The Katz and Hillier formulas were used to correct serum sodium levels for hyperglycemia (22, 23). Serum sodium was measured using indirect potentiometry. Primary outcomes included length of stay in the hospital, length of mechanical ventilation time and elevated inflammatory markers.

### Statistical analysis

Descriptive statistics were expressed as medians with 25th−75th percentiles reported for continuous variables and as counts with frequencies (%) for categorical variables. Mann–Whitney *U*-tests were used to compare continuous variables while Pearson's chi^2^ test or Fisher's exact test was used to compare categorical variables. Due to sample size limitations, adjusted models measuring evaluating hyponatremia. Logistic regression models assessed associations of the hyponatremia, the independent variable, with mortality, length of stay and need for mechanical ventilation. Variables included within the models were determined based on *a priori* variables of interest and potential confounders, including age, sex, race, PICU stay and primary diagnosis (COVID-19 or MIS-C), were added as clusters in a forward stepwise manner due to sample size limitation. Linear regression models were utilized to evaluate the association of hyponatremia with length of stay and levels of inflammatory markers. Standard assumptions of Gaussian residuals and quality of variance were tested. If the standard assumptions were not met, a transformation (i.e., natural logarithm, square route) was performed. Variables included in the models were determined based on *a priori* variables of interest and potential confounders and added as clusters in a forward stepwise manner due to sample size limitations. Missing values were rare in the dataset and were not imputed. Results were considered statistically significant at the *p* < 0.05 level of significance. All analyses were performed using Stata 15.3 and JASP 0.16.

## Results

### Baseline results

Of the 168 children in the study cohort, 95 (56%) were admitted for acute COVID-19 and 73 (43.5%) were admitted for MIS-C. Both patient populations had similar ages, gender, BMI *z*-scores, racial and ethnic demographics. Overall, 60 (35.7%) patients presented with hyponatremia, 95 (56%) with COVID and 73 (35.7%) with MIS-C (Table 1). Two patients re-classified from hyponatremia to eunatremia after correcting for hyperglycemia, using both the Katz and Hillier methods. No eunatremic patients were reclassified as hyponatremic. A larger proportion of MIS-C patients required intensive care; MIS-C patients also presented more often with fever, rash, and gastrointestinal symptoms as compared to patients with acute COVID-19 (*p*-value < 0.001). Admission sodium, potassium, calcium, and albumin were all significantly lower in the MIS-C population. Inflammatory markers such as fibrinogen, c-reactive protein (CRP), D-dimer, and treatment with vasoactive infusions were higher in the MIS-C cohort. MIS-C patients had a greater length of stay and presented with a higher prevalence of hyponatremia at admission, *p* = 0.007 and *p* < 0.001 respectively (Table 1). Two patients in the cohort died, both with acute Covid-19.

**Table 1:** Baseline demographic and clinical variables of children with acute COVID-19 vs. MIS-C.

Variables	Overall	Acute COVID-19	MIS-C	*P* value
	*N* = 168	*N* = 95	*N* = 73	COVID-19 vs. MIS-C
Age, year	8.6 (2.6, 13.3)	8.6 (1.5, 13.8)	8.7 (6.7, 12.8)	0.133
Male *N* (%)	94 (56.0)	48 (50.5)	46 (63.0)	0.106
Race *N* (%)				0.828
White	25 (14.9)	13 (13.7)	12 (16.4)	
Black/African American	37 (22.0)	22 (23.2)	15 (20.5)	
Asian	21 (12.5)	10 (10.5)	11 (15.1)	
Other/multiracial	76 (45.2)	45 (47.4)	31 (42.5)	
Unavailable/unknown	8 (4.8)	4 (4.2)	4 (5.5)	
Ethnicity *N* (%)				0.312
Hispanic/Latino	43 (24.7)	23 (24.2)	20 (27.4)	
Not Hispanic/Latino	115 (68)	66 (69.5)	49 (67.1)	
Other/unknown	10 (5.6)	6 (6.3)	4 (5.5)	
BMI *z*-score (*N* = 147) ^a^	0.270 (0.00, 1.84)	0.124 (0.00, 1.56)	0.484 (−0.51, 2.02)	0.869
Hospital type *N* (%)				
ICU admission	71 (42.3)	25 (26.3)	46 (63.0)	<0.001
Presenting symptoms *N* (%) (*N* = 147)				
Gastrointestinal	92 (62.6)	45 (47.4)	47 (90.4)	<0.001
Fever	117 (79.6)	67 (70.5)	50 (96.2)	<0.001
Cough	28 (19.0)	21 (22.1)	7 (13.5)	0.202
Rash	39 (26.5)	15 (15.6)	24 (46.2)	<0.001
Myalgias	12 (8.2)	6 (6.3)	6 (11.5)	0.346
Joint aches	5 (3.4)	4 (4.2)	1 (1.9)	0.656
Comorbid condition *N* (%) (*N* = 147)				
Asthma	11 (7.5)	7 (7.4)	4 (7.7)	1.0
Cancer	4 (2.7)	4 (4.2)	0 (0.0)	0.3
Congenital heart disease	9 (6.1)	7 (7.4)	2 (3.8)	0.493
Immunocompromised	4 (2.7)	4 (4.2)	0 (0.0)	0.71
Baseline SCr, mg/dl (*N* = 147) ^b,c^	0.58 (0.42, 0.72)	0.56 (0.39, 0.72)	0.61 (0.52, 0.72)	0.041
Admission SCr, mg/dl	0.45 (0.29, 0.66)	0.37 (0.24, 0.56)	0.56 (0.39, 0.76)	<0.001
Admission eGFR, ml/min/1.73 m^2^ (*N* = 147) ^d^	145 (101.6, 186.7)	146 (103.5,186.7)	141.4 (102.2, 183.9)	0.576
Admission lab values (mEq/L)				
Sodium	136 (133, 138)	137 (135, 138)	133 (131, 136)	<0.001
Bicarbonate	21 (19, 23)	21 (19, 23)	21 (18, 23)	0.0030.767
Albumin, mg/dl (*N* = 162)	3.9 (3.4, 4.3)	4.1 (3.7, 4.5)	3.6 (3.1, 3.9)	<0.001
While blood cells, mm^3^ (*N* = 162)	9.2 (6.3, 13.3)	8.7 (5.6, 13.7)	9.8 (7.1, 12.8)	0.297
Hemoglobin, g/dl (*N* = 162)	11.5 (10.3, 12.6)	11.6 (10.1, 12.7)	11.4 (10.5, 12.3)	0.884
Platelets, mm^3^ (*N* = 162)	221 (144.8, 295.8)	267 (187, 346.5)	173 (123, 262)	<0.001
LDH, U/L (*N* = 113) ^e^	321 (244, 422)	350 (290, 484)	301.5 (234.5, 366.5)	0.020
Fibrinogen, mg/dl (*N* = 119)	632 (505, 777)	507 (394, 608)	728 (626.5, 884.5)	<0.001
CRP, ug/ml (*N* = 132) ^f^	93.4 (29.8, 169.9)	29 (8.5, 88.1)	151.7 (87.5, 225.3)	<0.001
Ddimer, mcg/ml (*N* = 115)	845 (498, 1,998.5)	563 (372.5, 1,024)	1,276 (647.8, 2,321.3)	0.004
Outcomes				
LOS hospital (days) (*N* = 147)	3.96 (2.04, 7.44)	3.16 (1.69, 7.27)	4.67 (3.66, 7.34)	0.007
Mech ventilation (*N* = 146)	11 (7.4)	7 (7.4)	4 (7.4)	1.0
Acute kidney injury	21 (12.5)	7 (7.4)	14 (19.2)	0.022
Hyponatremia	60 (35.7)	16 (16.8)	44 (60.3)	<0.001
In hospital medication *N* (%)				
Vasopressors (*N* = 148) ^g^	32 (21.6)	6 (6.3)	26 (35.6)	<0.001

^a^ BMI, body mass index.

^b^ SCr, serum creatinine.

^c^ Baseline SCr was estimated from assumed eGFR 120 ml/min per 1.73 m^2^ using original Schwartz formula (24).

^d^ eGFR, estimated glomerular filtration rate.

^e^ LDH, lactate dehydrogenase.

^f^ CRP, C-reactive protein.

^g^ Use of dopamine/norepinephrin/epinephrine/vasopressin.

### Hyponatremia and acute COVID-19

The median age of children with acute-COVID-19 was 8.6 years (IQR 1.5–13.8) and over half were male. Sixteen (16.8%) patients presented with hyponatremia [132 mEq/L (IQR 129.8–134.0)] (Table 2). There were no significant differences in age, sex, race, ethnicity, or BMI *z*-score between children with and without hyponatremia. Moreover, there were no significant differences between presenting symptoms, comorbid conditions, hospital vasoactive infusion use, length of stay, or mechanical ventilation between the hyponatremic and eunatremic populations. Admission bicarbonate levels in hyponatremic patients were significantly lower than median admission bicarbonate levels in eunatremic patients (19 mEq/L (15.8–22.0) vs. 21 mEq/L (19.0–23.0), *p* = 0.016). Admission CRP levels were elevated in the hyponatremic population [84.3 ug/ml (IQR 40.7–155.8)] when compared to eunatremic patients [17.4 ug/ml (IQR 6.2–63)], *p* = 0.005 (Table 2). One of the two patients who died was hyponatremic on admission (serum sodium 129 mEq/L).

**Table 2:** MIS-C and COVID hyponatremia vs. Eunatremia.

Variables	Eunatremia	Hyponatremia	*P* value	Eunatremia	Hyponatremia	*P* value
	MIS-C	MIS-C	Eunatremia vs. hyponatremia	COVID	COVID	Eunatremia vs. hyponatremia
	*N* = 29	*N* = 44		*N* = 79	*N* = 16	
Age, year	10.8 (8.3, 14.8)	8.0 (4.7, 10.5)	<0.001	7.6 (1.5, 15.1)	8.8 (5.6, 11.3)	0.925
Male *N* (%)	20 (69.0)	26 (59.1)	0.392	40 (50.6)	8 (50.0)	0.963
Race *N* (%)			0.609			0.482
White	4 (13.8)	12 (16.4)		9 (11.4)	4 (25.0)	
Black/African American	5 (17.3)	10 (22.7)		17 (21.5)	5 (31.3)	
Asian	5 (17.3)	6 (13.6)		8 (10.1)	2 (12.5)	
Other/multiracial	12 (41.4)	19 (43.2)		40 (50.6)	5 (31.3)	
Unavailable/unknown	3 (10.3)	1 (2.3)		4 (5.1)	0 (0.0)	
Ethnicity *N* (%)			0.576			0.634
Hispanic/Latino	9 (31.0)	11 (25.0)		21 (26.6)	2 (12.5)	
Not Hispanic/Latino	18 (62.1)	31 (70.5)		53 (67.1)	13 (81.3)	
Other/unknown	2 (6.9)	2 (4.5)		5 (6.3)	1 (6.3)	
BMI *z*-score (*N* = 52) ^a^	1.74 (−0.18, 2.08)	0.223 (−0.73, 1.72)	0.185	0.12 (0.00, 1.62)	0.25 (−0.49, 1.16)	0.484
Hospital type *N* (%)						
ICU admission	17 (58.6)	29 (65.9)	0.528	22 (27.8)	3 (18.8)	0.451
Presenting symptoms *N* (%) (*N* = 52)						
Gastrointestinal	17 (85.0)	30 (93.8)	0.069	34 (43.0)	11 (68.8)	0.060
Fever	19 (95)	31 (96.9)	0.861	55 (69.6)	12 (75.0)	0.667
Cough	1 (5.0)	6 (18.8)	0.158	18 (22.9)	3 (18.8)	0.723
Rash	9 (45.0)	15 (46.9)	0.895	13 (16.5)	2 (12.5)	0.692
Myalgias	2 (10.0)	4 (12.5)	1.0	5 (6.3)	1 (6.3)	1.000
Joint aches	1 (5.0)	0 (0.0)	0.385	4 (5.1)	0 (0.0)	1.000
Comorbid condition *N* (%) (*N* = 52)						
Asthma	3 (15.0)	1 (3.1)	0.285	6 (7.6)	1 (6.3)	1.00
Cancer	0 (0.0)	0 (0.0)	0.3	4 (5.1)	0 (0.0)	1.00
Congenital heart disease	1 (5.0)	1 (3.1)	1.0	6 (7.6)	1 (6.3)	1.00
Immunocompromised	0 (0.0)	0 (0.0)	0.71	3 (3.8)	1 (6.3)	0.528
Baseline SCr, mg/dl (*N* = 52) ^b,c^	0.68 (0.60, 0.92)	0.56 (0.46, 0.64)	0.002	0.54 (0.37, 0.72)	0.58 (0.51, 0.66)	0.735
Admission SCr, mg/dl	0.65 (0.54, 0.80)	0.50 (0.31, 0.67)	0.036	0.35 (0.24, 0.52)	0.44 (0.28, 0.65)	0.498
Admission eGFR, ml/min/1.73 m^2^ (*N* = 52) ^d^	152 (103.1,175.1)	131.6 (100.8, 184)	0.52	146 (110.2,186)	147 (87.7, 190.9)	0.835
Admission lab values (mEq/L)						
Sodium	137 (136, 139)	132 (130, 133)	<0.001	137 (136, 139)	132 (129.8, 134)	<0.001
Bicarbonate	22 (21, 23)	20 (17.8, 22)	0.029	21 (19, 23)	19 (15.8, 22.0)	0.016
Albumin, mg/dl	3.7 (3.3, 4.1)	3.5 (3.1, 3.8)	0.122	4.1 (3.8, 4.5)	4.0 (3.6, 4.2)	0.083
While blood cells, mm^3^	9.9 (6.4, 12.2)	9.7 (7.2, 12.8)	0.727	8.9 (5.4, 14.0)	7.8 (7.0, 10.6)	0.962
Hemoglobin, g/dl	11.9 (11.0, 13.2)	11.1 (10.3, 12.0)	0.012	11.6 (10.2, 12.7)	11.5 (9.8, 12.3)	0.696
Platelets, mm^3^	184 (125, 262)	170 (121, 262.8)	0.600	271 (198, 347.8)	249 (175, 304)	0.623
LDH, U/L (*N* = 64) ^e^	295 (215, 368)	306 (244.5, 360.5)	0.715	369.5 (285, 524)	301 (291, 405)	0.296
Fibrinogen, mg/dl (*N* = 68)	745 (688.3, 911)	712.5 (600, 865.3)	0.187	506 (387, 608)	511 (419, 575)	0.713
CRP, ug/ml ^f^	156 (83, 201.3)	145.7 (87.5, 226)	0.910	17.4 (6.2, 63)	84.3 (40.7, 156)	0.005
Ddimer, mcg/ml (*N* = 68)	966 (494.5, 2,600)	1,288 (737, 1,933.8)	0.741	528 (351, 880)	845 (542, 2,321)	0.130
Outcomes						
LOS hospital (days) (*N* = 52)	4.79 (4.00, 8.03)	4.67 (3.56, 7.06)	0.592	2.83 (1.60, 7.27)	3.92 (2.13, 7.57)	0.149
Mech Ventilation (*N* = 54)	3 (14.3)	1 (3.0)	0.287	6 (7.6)	1 (6.3)	1.000
Acute kidney injury	4 (13.8)	10 (22.7)	0.343	4 (5.1)	3 (18.8)	0.090
Echocardiogram						
LVEF (*N* = 67)	55 (47.5, 60.25)	53 (48.5, 60)	0.726			
Systolic dysfunction	14 (48.3)	22 (50.0)	0.885			
Coronary artery dilation	24 (82.8)	39 (88.6)	0.475			
In-hospital medication *N* (%)						
Vasopressors (*N* = 54) ^g^	9 (42.9)	17 (51.5)	0.535	5 (6.58)	1 (6.25)	1.00

^a^ BMI, body mass index.

^b^ SCr, serum creatinine.

^c^ Baseline SCr was estimated from assumed eGFR 120 ml/min per 1.73 m^2^ using original Schwartz formula (24).

^d^ eGFR, estimated glomerular filtration rate.

^e^ LDH, lactate dehydrogenase.

^f^ CRP, C-reactive protein.

^g^ Use of dopamine/norepinephrin/epinephrine/vasopressinn.

### Hyponatremia and MIS-C

The median age of children with MIS-C was 8.7 years (IQR 6.7–12.8) with slightly more than half being male. Forty-four (60.3%) patients with MIS-C presented with hyponatremia. MIS-C patients with hyponatremia had a median age of 8.0 (IQR 4.7, 10.5); this was significantly younger than the eunatremic population with a median age of 10.8 (IQR 8.3, 14.8), *p* < 0.001 (Table 2). There were no significant differences in sex, race, ethnicity, and BMI *z*-score between children with and without hyponatremia. There were no significant differences in presenting symptoms, comorbid conditions and in hospital vasoactive medication usage between the hyponatremic and eunatremic populations. Echocardiographic data was available and analyzed for all MIS-C patients (*n* = 73) (Table 2). There were no significant differences between hyponatremic and eunatremic populations for left ventricle ejection fraction (LVEF), systolic dysfunction status, or coronary artery dilation.

### Hyponatremia in the overall

Hyponatremic patients had lower admission calcium, bicarbonate, albumin, and platelet levels as compared to eunatremic patients. However, hyponatremic patients presented with significantly higher rates of gastrointestinal symptoms (*p* < 0.001), fever (*p* = 0.036), and D-dimer levels (*p* = 0.014) (Table 3). Hyponatremic patients had an elevated median CRP level as compared to eunatremic patients (133.4 ug/ml (IQR 80.8, 210.9), while eunatremic patients had a median CRP level of 56.6 ug/ml (IQR 13.1, 152.8), *p* < 0.001 (Table 3). There were no significant differences in total length of stay or mechanical ventilation usage between the hyponatremic and eunatremic populations. Of the total cohort, 71 (42.2%) were admitted to the pediatric intensive care unit (PICU). The median age of the PICU population was (10.8 (IQR 6.4, 14.7) vs. 7.4 (IQR 1.9, 11), *p* = 0.002) (Supplementary Table S1). PICU patients had higher prevalence of hyponatremia, elevated admission CRP levels, and elevated D-dimer; they also had lower albumin, potassium, calcium, sodium, and platelet count (Supplementary Table S1). The elevated CRP levels and diminished albumin level of hyponatremic vs. eunatremic patients were maintained in analysis of only patients admitted to the intensive care unit (Supplementary Table S2). In addition, within the PICU population, those with hyponatremia were more likely to have gastrointestinal symptoms, in hospital vasoactive medication usage and elevated bicarbonate levels (Supplementary Table S2).

**Table 3:** Demographic and clinical characteristic of children with hyponatremia vs. eunatremia.

Variables	Overall	Eunatremia	Hyponatremia	*P* value
	*N* = 168	*N* = 108	*N* = 60	Eunatremia vs. hyponatremia
Age, year	8.6 (2.6, 13.3)	9.8 (2.0, 14.9)	8.1 (4.7, 10.6)	0.270
Male *N* (%)	94 (56.0)	60 (55.6)	34 (56.7)	0.889
Race *N* (%)				0.407
White	25 (14.9)	13 (12.0)	12 (20.0)	
Black	37 (22.0)	22 (20.4)	15 (25.0)	
Asian	21 (12.5)	13 (12.0)	8 (13.3)	
Other/multiracial	77 (45.8)	52 (48.1)	24 (40.0)	
Ethnicity *N* (%)				0.534
Hispanic/Latino	43 (24.7)	30 (27.8)	13 (21.7)	
Other/unknown	10 (5.6)	7 (6.5)	3 (5.0)	
BMI *z*-score (*N* = 147) ^a^	0.270 (0.00, 1.84)	0.270 (0.00, 1.89)	0.23 (−0.67, 1.63)	0.227
Hospital type *N* (%)				
ICU admission	71 (42.3)	39 (36.1)	32 (53.3)	0.030
Presenting symptoms *N* (%) (*N* = 147)				
Gastrointestinal (*N* = 147)	92 (62.6)	51 (51.5)	41 (85.4)	<0.001
Fever (*N* = 147)	117 (79.6)	74 (74.7)	43 (89.6)	0.036
Cough (*N* = 147)	28 (19.0)	19 (19.2)	9 (18.8)	0.949
Rash (*N* = 147)	39 (26.5)	22 (22.2)	17 (35.4)	0.089
Myalgias (*N* = 147)	12 (8.2)	7 (7.1)	5 (10.4)	0.528
Joint aches (*N* = 147)	5 (3.4)	5 (5.1)	0 (0.0)	0.173
Comorbid condition *N* (%) (*N* = 147)				
Asthma	11 (7.5)	9 (9.1)	2 (4.2)	0.505
Cancer	4 (2.7)	4 (4.0)	0 (0.0)	0.304
Congenital heart disease	9 (6.1)	7 (7.1)	2 (4.2)	0.718
Immunocompromised	4 (2.7)	3 (3.0)	1 (2.1)	1.0
Baseline SCr, mg/dl (*N* = 147) ^b,c^	0.58 (0.42, 0.72)	0.61 (0.39, 0.73)	0.57 (0.46, 0.66)	0.680
Admission SCr, mg/dl	0.45 (0.29, 0.66)	0.42 (0.28, 0.66)	0.48 (0.31, 0.66)	0.326
Admission eGFR, ml/min/1.73 m^2^ (*N* = 147) ^d^	145 (101.6, 186.7)	147 (108.7,186.2)	137.1 (92.5, 186.9)	0.444
Admission lab values (mEq/L)				
Sodium	136 (133, 138)	137 (136, 139)	132 (130, 133)	<0.001
Bicarbonate	21 (19, 23)	21 (19, 23)	19.5 (17, 22)	0.006
Albumin, mg/dl (*N* = 162)	3.9 (3.4, 4.3)	4.1 (3.6, 4.5)	3.6 (3.1, 3.9)	<0.001
While blood cells, mm^3^ (*N* = 162)	9.2 (6.3, 13.3)	9.3 (5.5, 13.7)	9.1 (7.1, 12.6)	0.537
Hemoglobin, g/dl (*N* = 162)	11.5 (10.3, 12.6)	11.6 (10.5, 12.8)	11.1 (10.2, 12.1)	0.107
Platelets, mm^3^ (*N* = 162)	221 (144.8, 295.8)	242 (163, 328)	178 (130, 271)	0.026
LDH, U/L (*N* = 113) ^e^	321 (244, 422)	346 (241, 444)	303 (245, 367)	0.218
Fibrinogen, mg/dl (*N* = 119)	632 (505, 777)	620 (479, 732)	656.5 (535, 848)	0.096
CRP ug/ml (*N* = 132) ^f^	93.4 (29.8, 169.9)	56.6 (13.1, 152.8)	133.4 (80.8, 210.9)	<0.001
Ddimer, mcg/ml (*N* = 115)	845 (498, 1,998.5)	606 (366, 1,973)	1,279 (688, 2,006.5)	0.014
MIS-C	73 (43.45)	29 (26.85)	44 (73.33)	<0.001
Outcomes				
LOS hospital (days) (*N* = 147)	3.96 (2.04, 7.44)	3.71 (1.73, 7.71)	4.58 (3.08, 7.06)	0.075
Mech ventilation (*N* = 146)	11 (7.4)	9 (9.0)	2 (4.1)	0.341
Acute kidney injury	21 (12.5)	8 (7.4)	13 (21.7)	0.013
In-hospital medication *N* (%)				
Vasopressors (*N* = 148) ^g^	32 (21.6)	14 (14.4)	18 (35.3)	41

^a^ BMI, body mass index.

^b^ SCr, serum creatinine.

^c^ Baseline SCr was estimated from assumed eGFR 120 ml/min per 1.73 m^2^ using original Schwartz formula (24).

^d^ eGFR, estimated glomerular filtration rate.

^e^ LDH, lactate dehydrogenase.

^f^ CRP, C-reactive protein.

^g^ Use of dopamine/norepinephrin/epinephrine/vasopressin.

### Hyponatremia and outcomes

There was no association of hyponatremia with LOS or mechanical ventilation in bivariate associations or in regression models. However, in linear regression models, adjusted for age, sex, race, ICU stay and primary diagnosis (COVID-19 or MIS-C), the square root CRP was significantly increased (β = 1.79: 95% CI: 0.22–3.36) and the albumin level was 0.22 mg/dl lower (β = −0.22: 95% CI: −0.42–−0.01), as compared to those with eunatremia (Table 4, Supplementary Table S3).

**Table 4:** Adjusted models associations of hyponatremia with CRP and albumin^c^.

Variable	β (95% CI)^a^	*P* value
Square root of CRP^b^		
Hyponatremia	1.79 (0.22–3.36)	0.026
Albumin		
Hyponatremia	−0.22 (−0.42–−0.01)	0.039

^a^ CI, confidence interval.

^b^ CRP, C-reactive protein.

^c^ The model was adjusted by ICU status, MIS-C status, age, gender and race.

## Discussion

This study adds to the current literature about the epidemiology of pediatric hyponatremia in COVID19 and MIS-C and is, to our knowledge, the first to look at the associations of hyponatremia in pediatric acute COVID-19 and MIS-C. Hyponatremia was present on admission in over a third of the cohort (35.7%), 44 (60.3%) of the patients with MIS-C and 16 (16.8%) of children with acute COVID-19. Contrary to our hypothesis, hyponatremia on admission was not associated greater length of stay or need for mechanical ventilation (25). There was a significant association between hyponatremia and higher CRP and lower serum albumin levels which may suggest increased inflammation in children with hyponatremia and COVID19/MISC.

The incidence of hyponatremia in over a third (37.5%) of our cohort was not dissimilar to reported incidences of hyponatremia in adults with COVID19. Hirsch et al. evaluated a cohort of 9,943 adults with COVID-19 from Northwell Health and reported a prevalence of admission hyponatremia in over a third of hospitalizations (16). The incidence of hyponatremia was higher than that expected in a general hospital cohort. Another European study estimated the incidence of COVID19 related hyponatremia in adults as 16.8% higher as compared to control patients without COVID19 (13). While reports from the US and Europe differed, they almost all report a higher incidence of hyponatremia in COVID19 patients. Looking at the children in this study's cohort with COVID19, the incidence of hyponatremia is just 16.8% and approximates the reported prevalence of hyponatremia in hospitalized children (26). The lower incidence of hyponatremia in pediatric COVID-19 patients may be in part due to the fact that children have less severe disease manifestations as compared to adults (14).

The etiology of hyponatremia in pediatric COVID-19 may be caused by decreased effective circulating blood volume, extracellular fluid volume depletion or SIADH (27, 28). Children with hyponatremia overall had more GI issues. Hypovolemic hyponatremia is often attributed to gastrointestinal sodium loss through vomiting and diarrhea, although SAR-CoV-2 patients with acute gastroenteritis were found to have a lower risk of hyponatremia when compared to other pathogens (29, 30). In addition, patients with pain, nausea or pneumonia related to COVID-19 can develop SIADH through the non-osmotic release of arginine vasopressin induced by increased levels of cytokines and inflammation (31, 32). Children with hyponatremia had higher CRP and hypoalbuminemia, both markers for inflammation, as compared to eunatremic patients which may suggest potentiation of inflammation and in some, SIADH.

A meta-analysis of MIS-C studies reported hyponatremia rates as high as 80.8% (20). Similarly, this study found hyponatremia rates in children with MISC (60.3%) analogous to adults with severe COVID-19. This may be due to the similarity between the pathophysiology of MISC and the inflammatory response of adults with severe COVID-19 (16). Given the severity of presentation in MIS-C it is not surprising that the majority (*n* = 46, 63%) of our MIS-C population were admitted to the intensive care unit (9). Throughout the pandemic this remained true as the clinical severity of MIS-C was similar across Alpha, Delta and Omricon SARS-CoV-2 strains (33).

The higher rates of hyponatremia in MIS-C patients can be due to the aforementioned suggested mechanisms, of dehydration through GI loss and SIADH from the high levels of inflammation as discussed above. Inflammation as discussed above may also play a key role to be most similar to MIS-C. MIS-C shares several clinical features with both KD and Toxic Shock Syndrome, two phenomena associated with higher rates of hyponatremia (21). In a study on Kawasaki disease, elevated CRP was associated with elevated IL-6, elevated IL-1β, and subsequently rates of hyponatremia (34).

The association between COVID-19 and hyponatremia in adult populations demonstrated increased length of hospital stay, need for mechanical ventilation, and mortality in hyponatremic patients (16). Our study did not find any relationship between hyponatremia and the aforementioned outcomes in the COVID-19 or MIS-C population. Given the low mortality rates risk of mortality was not evaluated but it should be noted that we had a small sample size and thus are limited to test and measure strong associations.

The analysis suggests that hyponatremia was associated with elevated CRP levels and lower albumin levels, markers of inflammation. CRP is a non-specific protein induced by IL-6 in the liver and is a sensitive biomarker for inflammation and tissue damage (35). The pathogenesis of COVID-19 involves a potent inflammatory response, and CRP is an early predictor of severe COVID-19 and extremely elevated CRP levels may precede or reflect cytokine storm (36–38).

Similarly, in adults, hypoalbuminemia is associated with severe COVID-19 and researchers have found an inverse relationship between the levels of albumin and the risk of death in COVID-19 patients (39). While the mechanism behind hypoalbuminemia in COVID-19 is likely multifactorial and low albumin levels were seen predominately in adults with severe COVID-19 suggesting a phenomenon beyond hepatocellular dysfunction (39). Inflammation increases capillary permeability, leading to serum albumin release into interstitial space (40). Hypoalbuminemia often leads to lowered plasma oncotic pressure which leads to antidiuretic hormone release which can contribute to hyponatremia as well.

There are several limitations to this study. Due to the retrospective nature of the study, causality cannot be determined. Our sample size was small and thus conclusions should be made with caution. Moreover, serum sodium was measured using indirect potentiometry which is frequently utilized yet less accurate then direct potentiometry in pediatric populations (41–43). However, the study corrected for hyperglycemia the most common cause of pseudohyponatremia. Indirect potentiometry measurements are falsely depressed as total protein increases, as seen in our study with albumin, which could have resulted in an overdiagnosis of hyponatremia (44). Furthermore, the study lacked the data and serotyping of viral strains (alpha vs. delta variant) and thus models were not unable to adjust for strains. A strength of our study is that it represents patients from the greater New York City area at the epicenter of the COVID-19 outbreak, thus including a diverse racial, ethnic, and socioeconomic population. As noted above it took into account common confounders such as hyperglycemia and adjusted for these findings using validated equations.

In conclusion, hyponatremia occurred in over a third of children with acute COVID-19 and MIS-C. There was no association between hyponatremia and length of hospital stay or need for mechanical ventilation. Yet hyponatremia was associated with a higher serum CRP and lower serum albumin, which may reflect increased state of inflammation. Serum sodium on admission may be a useful and easily accessible marker to distinguish which children with COVID19 and MISC develop a higher immune response. Further prospective studies in larger cohorts are needed is needed to evaluate the prognostic use of admission hyponatremia, validated with serum cytokine levels in children with acute COVID-19 and MIS-C.

## Data Availability

The datasets presented in this article are not readily available because of PHI and is sensitive to the patients involved. Requests to access the datasets should be directed to abasalely@northwell.edu.
