# Supplementary material for: The prevalence and outcomes of hyponatremia in children with COVID-19 and multisystem inflammatory syndrome in children (MIS-C)
Source: Front Pediatr. 2023 Sep 7;11:1209587. doi: 10.3389/fped.2023.1209587 (PMC10513389; doi:10.3389/fped.2023.1209587)
Supplement: Supplementary file 1 [file Table1.doc]

Supplemental Table 1: PICU Admission vs No PICU Admission

| Variables | Overall  N = 168 | No PICU  N = 97 | PICU  N =71 | P value  PICU vs. No PICU |
| --- | --- | --- | --- | --- |
| Age, year | 8.6 (2.6, 13.3) | 7.4 (1.9, 11.0) | 10.8 (6.4, 14.7) | 0.002 |
| Male N (%) | 94 (56.0) | 53 (54.6) | 41 (57.7) | 0.689 |
| Race N (%)  White  Black/African American  Asian  Other/Multiracial  Unavailable/Unknown | 25 (14.9)  37 (22.0)  21 (12.5)  76 (45.2)  8 (4.8) | 17 (17.5)  19 (19.6)  10 (10.3)  46 (47.4)  4 (4.1) | 8 (11.3)  18 (25.4)  11 (15.5)  30 (42.3)  4 (5.6) | 0.828 |
| Ethnicity N (%)  Hispanic/Latino  Not Hispanic/Latino  Other/Unknown | 43 (24.7)  115 (68)  10 (5.6) | 28 (28.9)  63 (64.9)  6 (6.2) | 15 (21.1)  52 (73.2)  4 (5.6) | 0.312 |
| BMI z-score (N=147) | 0.270 (0.00, 1.84) | 0.0 (-0.01, 1.91) | 0.81 (0.0, 1.81) | 0.394 |
| Presenting Symptoms N (%) (N=147)  Gastrointestinal  Fever  Cough  Rash  Myalgias  Joint aches | 92 (62.6)  117 (79.6)  28 (19.0)  39 (26.5)  12 (8.2)  5 (3.4) | 53 (58.9)  71 (78.9)  15 (16.7)  20 (22.2)  4 (4.4)  3 (3.3) | 39 (90.4)  46 (80.7)  13 (22.8)  19 (33.3)  8 (14.0)  2 (3.5) | 0.245  0.790  0.356  0.137  0.061  1.00 |
| Comorbid Condition N (%) (N=147)  Asthma  Cancer  Congenital Heart Disease  Immunocompromised | 11 (7.5)  4 (2.7)  9 (6.1)  4 (2.7) | 6 (6.7)  1 (1.1)  5 (5.6)  2 (2.2) | 5 (8.8)  3 (5.3)  4 (7.0)  2 (3.5) | 0.750  0.299  0.735  0.641 |
| Baseline SCr, mg/dL (N=147) ^a^  Admission SCr, mg/dL  Admission eGFR, mL/min/1.73m^2^ (N=147) | 0.58 (0.42, 0.72)  0.45 (0.29, 0.66)  145 (101.6, 186.7) | 0.54 (0.39, 0.66)  0.37 (0.24, 0.50)  159.7 (116.6,191) | 0.66 (0.50, 0.75)  0.59 (0.41, 0.82)  121.6 (91.5, 157.2) | 0.004  <0.001  <0.001 |
| Admission Lab Values (mEq/L)  Sodium  Bicarbonate | 136 (133, 138)  21 (19, 23) | 136 (134, 138)  21 (19, 23) | 135 (131.5, 137)  20 (18, 23) | <0.001  0.479 |
| Albumin, mg/dL (N=162) | 3.9 (3.4, 4.3) | 4.1 (3.9, 4.5) | 3.5 (3.0, 3.9) | <0.001 |
| While blood cells, mm^3^ (N=162)  Hemoglobin, g/dL (N=162)  Platelets, mm^3^ (N=162) | 9.2 (6.3, 13.3)  11.5 (10.3, 12.6)  221 (144.8, 295.8) | 9.2 (6.0, 12.5)  11.6 (10.3, 12.7)  264 (175, 346) | 9.3 (6.7, 14.0)  11.3 (10.4, 12.3)  182 (122, 265) | 0.389  0.484  <0.001 |
| LDH, U/L (N = 113)  Fibrinogen, mg/dL (N = 119)  CRP, ug/mL (N = 132)  Ddimer, mcg/mL (N = 115) | 321 (244, 422)  632 (505, 777)  93.4 (29.8, 169.9)  845 (498, 1998.5) | 326.5 (225.5, 407)  621 (481, 731)  66.7 (13.9, 118.4)  588 (423.3, 900) | 320 (260.5, 425)  656 (522.3, 820.8)  150.2 (54.4, 225.3)  1281 (651.5, 2362) | 0.476  0.226  <0.001  <0.001 |
| MIS-C | 73 (43.45) | 27 (27.84) | 46 (64.79) | <0.001 |
| Outcomes  LOS Hospital (days) (N=147)  Mech Ventilation (N=146)  Acute Kidney Injury  Hyponatremia | 3.96 (2.04, 7.44)  11 (7.4)  21 (12.5)  62 (36.9) | 2.87 (1.63, 4.67)  0 (0.0)  3 (3.1)  29 (29.9) | 7.71 (3.71, 14.79)  11 (19.3)  18 (25.4)  32 (45.1) | <0.001  <0.001  <0.001  0.028 |

BMI, body mass index; CRP, C-reactive protein; eGFR, estimated glomerular filtration rate; LDH, lactate dehydrogenase; SCr, serum creatinine.

Data are given as median (interquartile range) or as Count (percentage)

^a^Baseline SCr was estimated from assumed eGFR 120 ml/min per 1.73 m^2^ using original Schwartz formula.[25](https://www.ncbi.nlm.nih.gov/pmc/articles/PMC7927648/#bib25)

*Use of dopamine/norepinephrine/epinephrine/vasopressin
