# Supplementary material for: The prevalence and outcomes of hyponatremia in children with COVID-19 and multisystem inflammatory syndrome in children (MIS-C)
Source: Front Pediatr. 2023 Sep 7;11:1209587. doi: 10.3389/fped.2023.1209587 (PMC10513389; doi:10.3389/fped.2023.1209587)
Supplement: Supplementary file 2 [file Table2.doc]

Supplemental Table 2: PICU Hyponatremia vs PICU Eunatremia

| Variables | Overall PICU  N = 71 | Eunatremia  N = 39 | Hyponatremia  N =32 | P value  Eunatremia vs Hyponatremia |
| --- | --- | --- | --- | --- |
| Age, year | 10.8 (6.4, 14.7) | 13.08 (7.0, 16.4) | 8.6 (6.4, 12.5) | 0.114 |
| Male N (%) | 41 (57.7) | 22 (56.4) | 19 (59.3) | 0.801 |
| Race N (%)  White  Black  Asian  Other/Multiracial | 8 (11.3)  18 (25.4)  11 (15.5)  30 (40.3) | 3 (7.7)  12 (30.8)  5 (12.8)  16 (41.0) | 5 (15.6)  6 (18.8)  6 (18.8)  14 (43.8) | 0.547 |
| Ethnicity N (%)  Hispanic/Latino  Other/Unknown | 15 (21.1)  4 (5.6) | 8 (20.5)  3 (7.7) | 7 (21.9)  1 (3.1) | 0.637 |
| BMI z-score (N = 57) | 0.881 (0.00, 1.81) | 0.896 (0.00, 1.83) | 0.464 (-0.51, 1.43) | 0.073 |
| Presenting Symptoms N (%)  Gastrointestinal (N= 57)  Fever (N= 57)  Cough (N= 57)  Rash (N= 57)  Myalgias (N= 57)  Joint aches (N= 57) | 39 (68.4)  46 (80.7)  13 (22.8)  19 (33.3)  8 (14.0)  2 (3.5) | 17 (51.5)  24 (72.7)  9 (27.3)  10 (30.3)  4 (12.1)  2 (6.1) | 22 (91.7)  22 (91.7)  4 (16.7)  9 (37.5)  4 (16.7)  0 (0.0) | 0.001  0.074  0.346  0.569  0.709  0.504 |
| Comorbid Condition N (%)  Asthma (N= 57)  Cancer (N= 57)  Heart Disease (N= 57)  Immunocompromised (N= 57) | 5 (8.8)  3 (5.3)  4 (7.0)  2 (3.5) | 4 (12.1)  3 (9.1)  2 (6.1)  2 (6.1) | 1 (4.2)  0 (0.0)  2 (8.3)  0 (0.0) | 0.385  0.256  1.00  0.504 |
| Baseline SCr, mg/dL (N = 57) ^a^  Admission SCr, mg/dL  Admission eGFR, mL/min/1.73m^2^ (N = 57) | 0.66 (0.50, 0.75)  0.59 (0.41, 0.82)  121.6 (91.5, 157.2) | 0.72 (0.45, 0.89)  0.65 (0.38, 0.82)  127.8 (99.1,161.6) | 0.61 (0.53, 0.67)  0.59 (0.46, 0.81)  112 (64.2, 138.2) | 0.310  0.171  0.104 |
| Admission Lab Values (mEq/L)  Sodium  Bicarbonate | 135 (131.5, 137)  20 (18, 23) | 137 (136, 139)  21 (19, 24) | 131 (129.8, 133)  19 (17, 21) | <0.001  0.017 |
| Albumin, mg/dL | 3.5 (3.0, 3.9) | 3.6 (3.2, 4.1) | 3.4 (2.8, 3.7) | 0.032 |
| While blood cells, mm^3^  Hemoglobin, g/dL  Platelets, mm^3^ | 9.3 (6.7, 14.0)  11.3 (10.4, 12.3)  182 (122, 265) | 9.3 (5.4, 14.2)  11.5 (10.5, 13.3)  204 (130, 280.5) | 9.5 (7.5, 13.5)  11.1 (10.2, 11.7)  163 (118.5, 224.3) | 0.412  0.474  0.256 |
| LDH, U/L (N = 67)  Fibrinogen, mg/dL (N = 68)  CRP, ug/mL (N = 69)  Ddimer, mcg/mL (N = 67) | 320 (260.5, 425)  656 (522.3, 820.8)  150.2 (54.4, 225.3)  1281 (651.5, 2362) | 365 (283.5, 496)  635.5 (479.3, 768)  88.9 (21.9, 201.3)  1093 (371, 2967) | 309.5 (244.8, 350)  696 (573.8, 847.3)  180.6 (117.2, 266.1)  1424 (842.8, 2111.8) | 0.045  0.141  0.022  0.390 |
| MIS-C | 46 (64.79) | 17 (43.59) | 29 (90.63) | <0.001 |
| Outcomes  LOS Hospital (days) (N = 57)  Mech Ventilation (N= 57)  Acute Kidney Injury | 7.71 (3.71, 14.8)  11 (19.3)  18 (25.4) | 9.3 (4.6, 20.9)  9 (27.3)  7 (17.9) | 5.1 (3.65, 10.96)  2 (8.3)  11 (34.4) | 0.061  0.074  0.113 |
| In-Hospital Medication N (%)  Vasopressors* | 32 (45.1) | 14 (35.8) | 18 (56.25) | 0.006 |

BMI, body mass index; CRP, C-reactive protein; eGFR, estimated glomerular filtration rate; LDH, lactate dehydrogenase; SCr, serum creatinine.

Data are given as median (interquartile range) or as Count (percentage)

^a^Baseline SCr was estimated from assumed eGFR 120 ml/min per 1.73 m^2^ using original Schwartz formula.[25](https://www.ncbi.nlm.nih.gov/pmc/articles/PMC7927648/#bib25)

*Use of dopamine/norepinephrine/epinephrine/vasopressin
