# Supplementary material for: The prevalence and outcomes of hyponatremia in children with COVID-19 and multisystem inflammatory syndrome in children (MIS-C)
Source: Front Pediatr. 2023 Sep 7;11:1209587. doi: 10.3389/fped.2023.1209587 (PMC10513389; doi:10.3389/fped.2023.1209587)
Supplement: Supplementary file 3 [file Table3.docx]

Supplemental Table 3: Adjusted Associations of Hyponatremia with CRP and Albumin

| **Variable** | **β (95% CI)** | **P value** |
| --- | --- | --- |
| **Square Root of CRP** |  |  |
| Hyponatremia  PICU  MIS-C  Age  Gender  Race | 1.79 (0.22 – 3.36)  2.12 (0.59 – 3.65)  4.63 (3.04 – 6.23)  0.09 (-0.06 –0.24)  0.54 (-0.94 – 2.03)  0.12 (-0.32 – 0.55) | 0.026  0.007  0.000  0.224  0.470  0.601 |
| **Albumin** |  |  |
| Hyponatremia  PICU  MIS-C  Age  Gender  Race | -0.22 (-0.42 – -0.01)  -0.54 (-0.73 – -0.34)  -0.23 (-0.44 – -0.03)  0.00 (-0.01 – 0.02)  0.00 (-0.19 – 0.19)  0.18 (-0.69 – 0.71) | 0.039  0.000  0.028  0.659  0.997  0.425 |

1. CI - confidence interval
2. CRP - C-reactive protein
